# Multimorbidity in Australia: Comparing estimates derived using administrative data sources and survey data

**DOI:** 10.1371/journal.pone.0183817

**Published:** 2017-08-29

**Authors:** Sanja Lujic, Judy M. Simpson, Nicholas Zwar, Hassan Hosseinzadeh, Louisa Jorm

**Affiliations:** 1 Centre for Big Data Research in Health, University of New South Wales, Sydney, Australia; 2 School of Public Health, University of Sydney, Sydney, Australia; 3 School of Public Health and Community Medicine, University of New South Wales, Sydney, Australia; Swinburne University of Technology, AUSTRALIA

## Abstract

**Background:**

Estimating multimorbidity (presence of two or more chronic conditions) using administrative data is becoming increasingly common. We investigated (1) the concordance of identification of chronic conditions and multimorbidity using self-report survey and administrative datasets; (2) characteristics of people with multimorbidity ascertained using different data sources; and (3) whether the same individuals are classified as multimorbid using different data sources.

**Methods:**

Baseline survey data for 90,352 participants of the 45 and Up Study—a cohort study of residents of New South Wales, Australia, aged 45 years and over—were linked to prior two-year pharmaceutical claims and hospital admission records. Concordance of eight self-report chronic conditions (reference) with claims and hospital data were examined using sensitivity (Sn), positive predictive value (PPV), and kappa (κ).The characteristics of people classified as multimorbid were compared using logistic regression modelling.

**Results:**

Agreement was found to be highest for diabetes in both hospital and claims data (κ = 0.79, 0.78; Sn = 79%, 72%; PPV = 86%, 90%). The prevalence of multimorbidity was highest using self-report data (37.4%), followed by claims data (36.1%) and hospital data (19.3%). Combining all three datasets identified a total of 46 683 (52%) people with multimorbidity, with half of these identified using a single dataset only, and up to 20% identified on all three datasets. Characteristics of persons with and without multimorbidity were generally similar. However, the age gradient was more pronounced and people speaking a language other than English at home were more likely to be identified as multimorbid by administrative data.

**Conclusions:**

Different individuals, with different combinations of conditions, are identified as multimorbid when different data sources are used. As such, caution should be applied when ascertaining morbidity from a single data source as the agreement between self-report and administrative data is generally poor. Future multimorbidity research exploring specific disease combinations and clusters of diseases that commonly co-occur, rather than a simple disease count, is likely to provide more useful insights into the complex care needs of individuals with multiple chronic conditions.

## Introduction

Chronic diseases are the leading cause of illness, disability and death, accounting for 68% of global [1] and 90% of all Australian deaths [2]. The prevalence of chronic conditions has been increasing over the past forty years [3], with the greatest growth seen in the concurrent presence of multiple chronic diseases (known as multimorbidity [4]), attributable to the ageing population, and advances in medical care and public health policy [5, 6]. One third of the Australian population [7] are estimated to have multimorbidity, with up to 80% of those aged 65 and over having three or more chronic conditions [8].

Appropriate and accurate measurement of the prevalence of chronic disease and multimorbidity is essential in order to monitor trends, estimate burden of disease, target preventive measures, and plan treatment and care delivery. A variety of data sources are used for monitoring, including population health surveys, disease registries and administrative databases (including primary health care, hospitalisation and medication data), with the use of the latter becoming increasingly common due to its efficient capture, ease of use and inexpensive nature [9]. However, the use of administrative data is not without drawbacks. These data have different levels of capture of chronic disease, and variable data quality [10–14]. Furthermore, not all patients with chronic diseases use hospital services, and even when they do, their admission record may not capture all of their conditions. Medication data, on the other hand, present a different set of challenges. In some instances, prescribed medications are clearly linked to the treatment of a specific chronic condition (e.g. insulin in diabetic patients). In other cases, medications may have multiple indications (e.g. β-blockers for heart failure and high blood pressure). The majority of Australian studies of multimorbidity have estimated multimorbidity using self-report data [15–19].

Research on comparative estimates of multimorbidity derived using different data sources is scarce. The majority of multimorbidity studies use only one dataset (for example [17–21]), with only a handful of studies [22–27] examined the difference in prevalence estimates between data sources. These studies found differences in estimates of multimorbidity, but these were largely attributable to differing study populations and numbers of conditions counted in the multimorbidity definition. Even when trying to standardise the multimorbidity definition by using the same list of chronic conditions [26] or comparing multimorbidity within the same sample [24], no study has examined whether the same people, using the same list of chronic conditions, are classified as multimorbid using different data sources.

The current study used record linkage of self-report survey data from a large cohort study with two sets of administrative data to compare ascertainment of common chronic conditions. Specific aims were to investigate: (1) the concordance of identification of chronic conditions and multimorbidity using self-report and administrative datasets; (2) the similarities and differences between people with multimorbidity ascertained using different datasets; and (3) whether the same individuals are classified as multimorbid using different data sources.

## Methods

### Data sources

#### The 45 and Up Study

The 45 and Up Study is a large-scale cohort study involving 266,950 men and women aged 45 years and over from the general population of New South Wales, Australia’s most populous state. The study is described in detail elsewhere [28]. In brief, participants in the 45 and Up Study were randomly sampled from the Department of Human Services (formerly Medicare Australia) enrolment database, which provides near complete coverage of the population. People 80+ years of age and residents of rural and remote areas were oversampled. Participants joined the Study by completing a baseline questionnaire between February 2005 and March 2009 and giving signed consent for linkage of their information to routine health databases [28]. Of those invited, about 18% participated and these comprised about 11% of the NSW population aged 45 and over [28]. The baseline questionnaire was modified over time in an attempt to better capture self-report or doctor-diagnosed common illnesses. There were three versions of the questionnaire. In version 1, asthma, hayfever and depression were not included. In versions 2 and 3 separate questions for asthma, hayfever and depression were present [29].

#### Pharmaceutical Benefits Scheme (PBS)

The PBS database contains information on Commonwealth subsidised claims for prescribed medicines listed on the Schedule of Pharmaceutical Benefits [30]. The main PBS beneficiaries include concession card holders (people aged 65 and over who meet an income test, people with disability, low income or facing a large burden of dependants) and general beneficiaries. Prior to 2012, only records for PBS-listed prescription medications for which a government subsidy was paid were recorded on the PBS data. This resulted in differential capture of prescribed medicines by concession card holders and general beneficiaries. Capture for concession card holders was complete, as all prescription medicines cost more that the concession threshold. However, PBS-medicines falling below the co-payment threshold for general beneficiaries were not captured in the PBS data. We therefore restricted our analyses to concession card holders only, to avoid potential incomplete capture of medicines dispensed to general beneficiaries. PBS data from 1 September 2005 to 20 December 2011 were linked deterministically to 45 and Up Study questionnaire data by the Sax Institute, using a unique identifier that was provided to the Department of Human Services (DHS). PBS data included date of dispensing, beneficiary status, PBS item code, Anatomical Therapeutic Chemical (ATC) code [31] and quantity supplied. Unless otherwise specified, the term medication data in the paper refers to the PBS data.

#### The NSW Admitted Patient Data Collection (APDC)

The APDC includes records of all public and private hospital admissions ending in a separation, i.e. discharge, transfer, type-change or death. Diagnoses are coded according to the Australian modification of the International Statistical Classification of Diseases and Related Problems 10^th^ Revision, ICD-10-AM [32]. Up to 55 diagnoses codes are recorded on the APDC, including the principal diagnosis and up to 54 additional diagnoses. The APDC from 1 July 2000 to 31 December 2013 was linked probabilistically to survey information from the 45 and Up Study by the NSW Centre for Health Record Linkage (www.cherel.org.au) using the ‘best practice’ protocol for preserving privacy [33]. Unless otherwise specified, the term hospital data in the paper refers to the APDC data.

### Study population

People aged 45 years and over were included in the analysis if they: (a) completed the 45 and Up Study baseline study questionnaire between 1 September 2007 and 2 March 2009; and (b) had a PBS record for any prescription medication within 2 years preceding the questionnaire date (longest lookback available). Only those with consistent PBS concession card holder status within the 2-year period were included. Information about hospitalisations for these participants was also obtained from the APDC data, restricted to the same 2-year period as the PBS data. People who answered version 1 of the 45 and Up Study baseline questionnaire (n = 37 088) were excluded, as it was not possible to ascertain self-report of doctor-diagnosed depression for these participants. Holders of a Department of Veterans’ Affairs health card (n = 6 299) were also excluded, as the PBS does not capture all the services provided to these individuals. A total of 90 352 people with consistent PBS concession card holder status were included in the analysis: 46,766 persons with claims data only (medication only); and 43 586 persons with both claims and hospitalisation records (medication + hospitalisation) (S1 Fig)

### Morbidity measures

A total of eight chronic conditions (hypertension, cancer, heart disease, stroke, diabetes, asthma, depression and Parkinson’s disease–hereafter referred to as ‘morbidities’) were selected for analysis, based on their availability in both self-report and administrative data.

Self-report morbidities were ascertained on the basis of responses to a single question “Has a doctor ever told you that you have (*name of condition*)?” in the baseline 45 and Up Study survey.

Morbidity in the hospital data was ascertained using ICD-10-AM codes in any of the 55 diagnosis fields (S1 Table). The initial list of eligible ICD-10 codes was obtained from the Charlson Index [34, 35] and Elixhauser Index [36, 37], and refined following advice from a clinical coder. If a condition was coded at least once in the 2-year lookback period, then a person was coded as having that condition in the hospital data.

Morbidity in the medication data was ascertained using ATC codes obtained from Rx-Risk-V [38, 39], published reports [40], and research articles [41–47]. A person was coded as having conditions of interest if a specific ATC code was present in the medication data at least twice in the 2-year lookback period, as it was expected that chronic condition medications would be used regularly. Where published literature had different ATC codes, we chose the codes that had the highest positive predictive value (S1 Table).

A count of conditions in each of the three datasets (self-report, medication and hospital) was created by summing the total number of chronic conditions, ranging from 0 to 8, as well as the total when stroke was excluded. Multimorbidity was defined as having two or more chronic conditions, which is the most commonly used definition in the literature [48]. Complex multimorbidity was defined as having three or more chronic conditions affecting three or more body systems [49].

### Statistical methods

#### Measures of agreement

Agreement between the three data sources was measured by estimating sensitivity (Sn), specificity (Sp), positive predictive value (PPV), negative predictive value (NPV) and Cohen’s kappa statistic (κ) using self-report morbidity measures as the reference. Sensitivity represents the percentage of those with a condition (according to self-report) who were correctly identified as having that condition in administrative data. Specificity represents the percentage of those without a self-report condition who did not have a condition in administrative data. PPV represents the percentage of those identified as having a condition of interest in the administrative data, who actually had the condition, according to self-report. NPV represents the percentage of those identified as not having a condition of interest in the administrative data, who did not have a condition according to the self-report. The kappa statistic (κ) represents the proportion agreement corrected for chance. Kappa values above 0.75 denote excellent agreement, 0.40 to 0.75 fair to good agreement and below 0.45 poor agreement [50].

#### Analysis

Logistic regression was used to model the odds of multimorbidity, within each dataset separately. All analyses were adjusted for age (categorised into four 10-year age groups and 85+) and sex, and adjusted odds ratios (aORs) and their corresponding 95% confidence intervals (CI) were calculated. A range of categorical variables were examined, including remoteness of residence, highest education attainment, Aboriginal or Torres Strait Islander origin, country of birth, language other than English spoken at home, household income and marital status. Information about these variables was obtained from the 45 and Up Study baseline questionnaire. All data management and analyses were conducted using SAS software, version 9.3 [51].

### Ethical approvals

Ethics approvals for this study were obtained from the NSW Population and Health Services Research Ethics Committee and the Aboriginal Health & Medical Research Ethics Committee. The conduct of the 45 and Up Study was approved by the University of New South Wales Human Research Ethics Committee.

## Results

### Sample characteristics

The sample comprised 90 352 participants, who all had a PBS record within the 2 years prior to joining the 45 and Up Study. Forty eight percent of participants also had a hospitalisation in the same timeframe. The mean age at survey completion was 70.2 years in the full sample, and 71.8 years among those with a hospital record. The median number of self-report conditions was 1, with hypertension being the most commonly reported. Other characteristics of the study population are presented in Table 1.

**Table 1:** Characteristics of the study population.

	Medication + Hospitalisation (N = 43,586)		Full sample (N = 90,352)	
Mean age, years (standard deviation)	71.8 (9.7)		70.2 (10.2)	
Median number of self-report chronic conditions (range)	1 (0–8)		1 (0–8)	
Median number of self-report chronic conditions (exc stroke) (range)	1 (0–7)		1 (0–7)	
Male sex, n (%)	20,509 (47.1)		40,032 (44.3)	
Born overseas, n (%)	10,300 (23.6)		22,575 (25.0)	
Speaks language other than English at home, n (%)	4,173 (9.6)		9,525 (10.5)	
Aboriginal or Torres Strait Islander, n (%)	410 (0.9)		904 (1.0)	
Self-report conditions:	% with this condition	% with 1+ other conditions	% with this condition	% with 1+ other conditions
Hypertension	48.7	70.6	46.2	63.8
Cancer	26.4	75.2	20.9	73.1
Heart disease	23.9	82.2	18.5	80.6
Stroke	7.3	88.8	5.6	87.8
Diabetes	15.2	87.7	13.6	84.4
Asthma	14.0	82.3	12.6	77.8
Depression	16.2	79.0	15.7	72.8
Parkinson’s	1.2	85.5	1.0	83.3

### Agreement measures

Table 2 summarises agreement measures for self-report and administrative data for all eight chronic conditions and multimorbidity definitions. Excellent levels of agreement beyond chance were only found for diabetes, in both medication and hospital datasets. Fair to good agreement was found for hypertension, asthma, depression and Parkinson’s disease in the medication data only. The agreement between self-report and hospital data was generally poor.

**Table 2:** Measures of agreement between self-report chronic conditions and administrative data, 2-year lookback.

Chronic condition	Sn (95% CI)	PPV (95% CI)	Sp (95% CI)	NPV (95%CI)	Kappa	Prevalence admin data	Prevalence self-report	Relative difference
**Hospital data (n = 43,586)**^3^								
Hypertension	34.5 (33.9–35.2)	72.7 (71.8–73.6)	87.7 (87.2–88.1)	58.5 (57.9–59.0)	0.23	23.1%	48.7%	-53%
Cancer	17.9 (17.2–18.6)	89.0 (87.7–90.2)	99.2 (99.1–99.3)	77.1 (76.7–77.5)	0.23	5.3%	26.4%	-80%
Heart disease	44.4 (43.4–45.3)	59.0 (57.9–60.1)	90.3 (90.0–90.6)	83.8 (83.4–84.2)	0.38	18.0%	23.9%	-25%
Stroke	13.2 (12.0–14.4)	74.7 (71.0–78.2)	99.7 (99.6–99.7)	93.6 (93.4–93.8)	0.21	1.3%	7.3%	-82%
Diabetes	78.6 (77.6–79.6)	86.1 (85.2–86.9)	97.7 (97.6–97.9)	96.2 (96.0–96.4)	0.79	13.8%	15.2%	-9%
Asthma	6.9 (6.3–7.5)	80.8 (77.2–83.9)	99.7 (99.7–99.8)	86.8 (86.5–87.1)	0.11	1.2%	14.0%	-91%
Depression	6.1 (5.6–6.7)	70.5 (66.7–73.9)	99.5 (99.4–99.6)	84.6 (84.2–84.9)	0.09	1.4%	16.2%	-91%
Parkinsons's	29.1 (25.4–33.0)	82.5 (76.5–87.3)	99.9 (99.9–99.9)	99.1 (99.0–99.2)	0.43	0.4%	1.2%	-65%
**MM**^1^	33.5 (32.8–34.2)	76.7 (75.8–77.6)	91.9 (91.6–92.3)	63.6 (63.1–64.1)	0.27	19.3%	44.2%	-56%
**Complex MM**^2^	7.4 (6.7–8.1)	67.9 (63.8–71.8)	99.6 (99.5–99.6)	89.7 (89.4–90.0)	0.11	1.2%	11.0%	-89%
**Medication data (n = 90,352)**^4^								
Hypertension	62.2 (61.7–62.6)	79.9 (79.4–80.3)	86.6 (86.3–86.9)	72.7 (72.4–73.1)	0.50	35.9%	46.2%	-22%
Cancer	4.5 (4.2–4.8)	47.6 (45.3–50.0)	98.7 (98.6–98.8)	79.6 (79.3–79.9)	0.05	2.0%	20.9%	-91%
Heart disease	67.9 (67.2–68.6)	35.3 (34.7–35.8)	71.7 (71.3–72.0)	90.8 (90.5–91.0)	0.29	35.7%	18.5%	93%
Stroke	64.1 (62.8–65.4)	16.0 (15.5–16.5)	80.0 (79.8–80.3)	97.4 (97.3–97.5)	0.18	22.5%	5.6%	300%
Diabetes	72.4 (71.7–73.2)	90.0 (89.4–90.5)	98.7 (98.6–98.8)	95.8 (95.6–95.9)	0.78	11.0%	13.6%	-19%
Asthma	65.4 (64.6–66.3)	57.3 (56.5–58.2)	93.0 (92.8–93.2)	94.9 (94.8–95.1)	0.55	14.4%	12.6%	14%
Depression	51.5 (50.7–52.3)	66.4 (65.5–67.3)	95.1 (95.0–95.3)	91.3 (91.1–91.5)	0.51	12.2%	15.7%	-22%
Parkinson’s	58.9 (55.7–62.0)	53.3 (50.3–56.4)	99.5 (99.4–99.5)	99.6 (99.5–99.6)	0.56	1.1%	1.0%	10%
**MM**^1^	60.4 (59.8–60.9)	62.5 (62.0–63.0)	78.4 (78.0–78.7)	76.8 (76.5–77.2)	0.39	36.1%	37.4%	-3%
**Complex MM**^2^	24.7 (23.7–25.6)	56.2 (54.5–57.8)	98.2 98.1–98.2)	93.2 (93.0–93.3)	0.31	3.8%	8.7%	-56%
**Medication or hospital data (n = 90,352)**^5^								
Hypertension	66.2 (65.8–66.7)	78.0 (77.6–78.5)	84.0 (83.7–84.3)	74.4 (74.0–74.7)	0.51	39.2%	46.2%	-15%
Cancer	13.1 (12.6–13.5)	68.5 (66.9–70.0)	98.4 (98.3–98.5)	81.0 (80.8–81.3)	0.16	4.0%	20.9%	-81%
Heart disease	73.1 (72.4–73.8)	35.7 (35.2–36.2)	70.0 (69.7–70.4)	92.0 (91.7–92.2)	0.31	38.0%	18.5%	105%
Stroke	65.8 (64.5–67.1)	16.3 (15.8–16.8)	79.9 (79.7–80.2)	97.5 (97.4–97.6)	0.19	22.6%	5.6%	303%
Diabetes	80.4 (79.7–81.1)	86.8 (86.2–87.4)	98.1 (98.0–98.2)	96.9 (96.8–97.1)	0.81	12.6%	13.6%	-7%
Asthma	65.8 (65.0–66.7)	57.3 (56.5–58.2)	92.9 (92.8–93.1)	95.0 (94.8–95.1)	0.55	14.5%	12.6%	15%
Depression	52.0 (51.1–52.8)	66.2 (65.3–67.0)	95.0 (94.9–95.2)	91.4 (91.1–91.6)	0.52	12.4%	15.7%	-21%
Parkinson’s	59.5 (56.3–62.6)	52.6 (49.6–55.6)	99.4 (99.4–99.5)	99.6 (99.5–99.6)	0.55	1.2%	1.0%	13%
**MM**^1^	80.4 (80.0–80.8)	59.7 (59.2–60.1)	65.1 (64.7–65.5)	83.8 (83.4–84.1)	0.43	39.2%	37.4%	5%
**Complex MM**^2^	72.0 (71.2–72.7)	35.9 (35.4–36.5)	78.5 (78.2–78.8)	94.4 (94.2–94.5)	0.36	5.1%	8.7%	-41%

Sn: sensitivity; PPV: positive predictive value; Sp: specificity; NPV: negative predictive value

^1^ Multimorbidity (MM): Presence of two or more chronic conditions, excluding stroke

^2^ Complex MM: Presence of three or more chronic conditions affecting 3 or more body systems, excluding stroke

^3^ Conditions ascertained from hospital diagnoses

^4^ Conditions ascertained from medication codes

^5^ Conditions ascertained from medication codes (for those without a hospitalisation), or medication or hospitalisation codes (for those with a hospitalisation)

**Fig 1:**
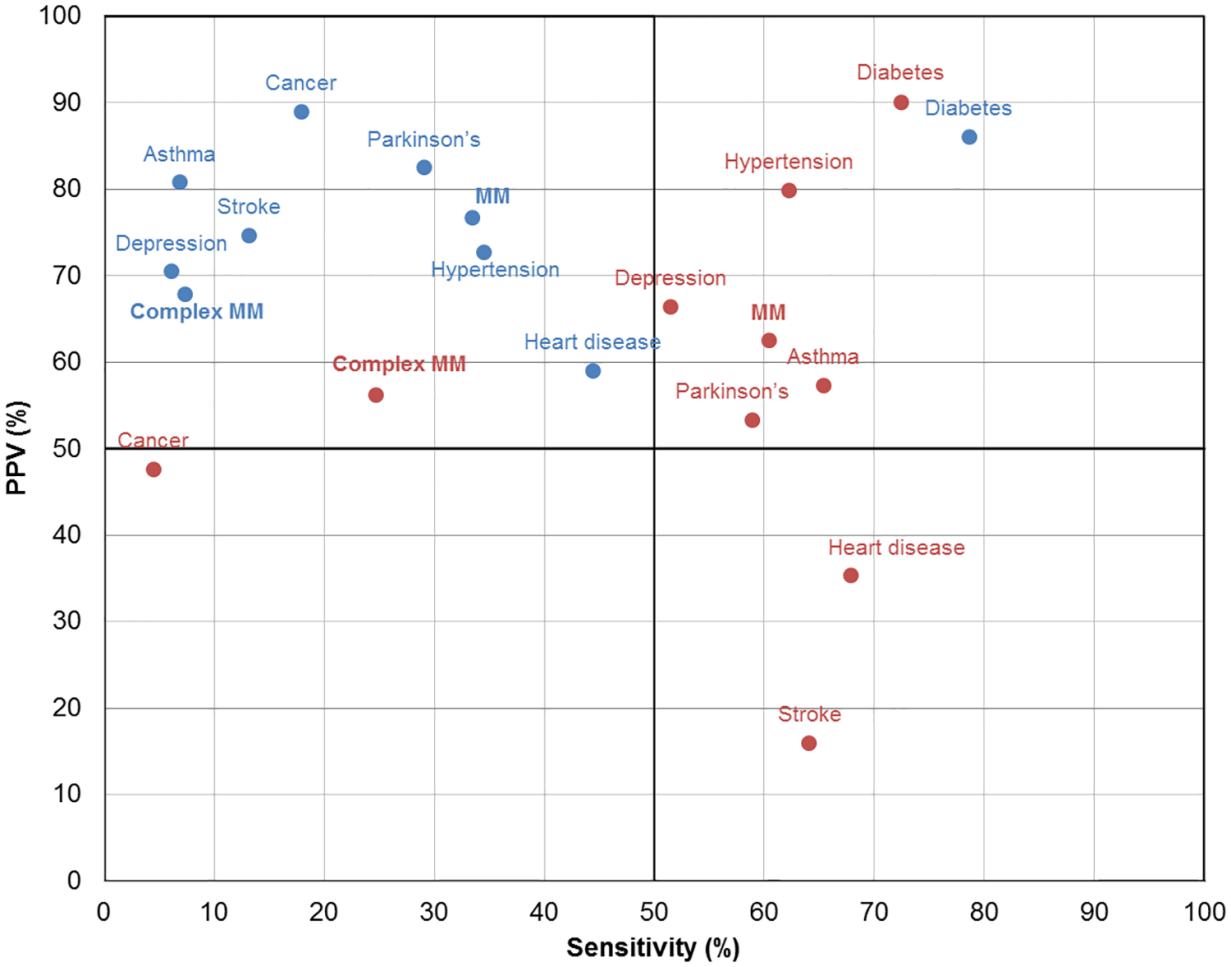
Agreement between self-report and administrative data sources. Blue circles–Hospital, Red circles–Medication. Abbreviations: MM–multimorbidity (2+ chronic conditions, excluding stroke); Complex MM–complex multimorbidity (3+ chronic conditions affecting 3 or more body systems, excluding stroke).

Except for cancer, sensitivity values were found to be higher in medication data (range 51.5% - 72.4%) than the hospital data (range 6.1% - 78.6%) (Fig 1). However, hospital data exhibited higher levels of PPV across all conditions, with the majority of PPVs higher than 70%. The highest PPV was for cancer (89%) in hospital data, and diabetes (90%) in medication data.

Prevalence of individual chronic conditions varied by data source, with hypertension identified in nearly 50% of the sample. Stroke prevalence estimates were found to be four times greater using medication data than self-report data (22.5% vs 5.6%), so stroke was excluded from the count of conditions in the remaining analyses.

### Prevalence of multimorbidity

The prevalence of multimorbidity in the study sample was highest using the self-report data (37.4% in the overall sample, 44.2% among those hospitalised), followed by medication data (36.1%) and hospital data (19.3%) (Table 2). The highest level of complex multimorbidity was found among hospitalised patients using the self-report multimorbidity definition (11%).

**Fig 2:**
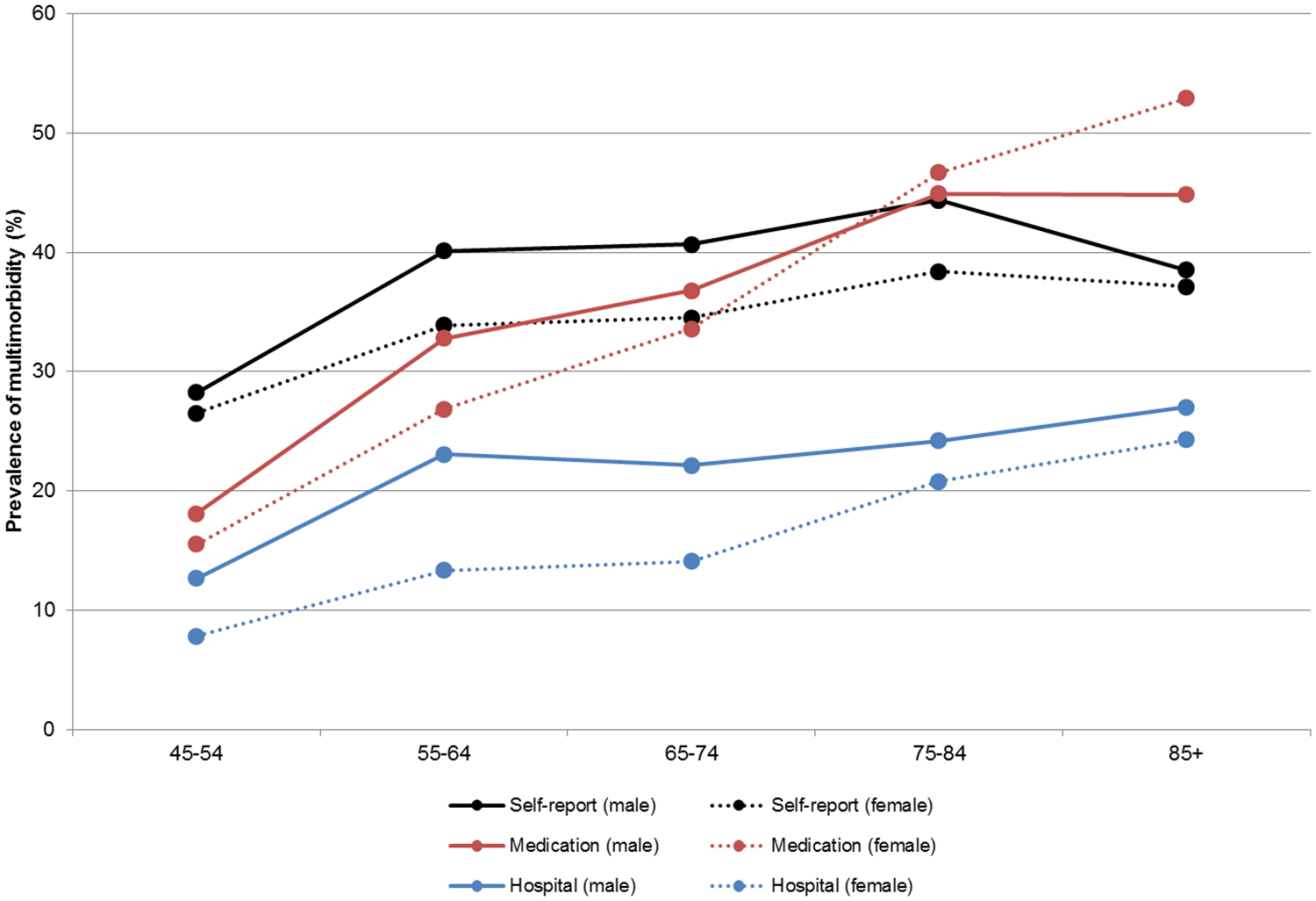
Prevalence of multimorbidity, by age group and data source. Black circles, solid line–Self-report (male); Black circles, broken line–Self-report (female); Red circles, solid line–Medication (male); Red circles, broken line–Medication (female); Blue circles, solid line–Hospital (male); Blue circles, broken line–Hospital (female).

The prevalence of multimorbidity was higher in males, and increased with age, using all three data definitions (Fig 2). For those aged under 75 years, the highest prevalence was found using self-report data. For people aged over 75 years, the estimates, particularly in women, were higher using medication data. The proportion of persons with multimorbidity was consistently lower in hospital data compared to the other two datasets.

Associations between multimorbidity and key demographic variables were found to be consistent between datasets, with some differences in the magnitudes of these relationships. The odds of multimorbidity were higher in people who were male, older, of Aboriginal or Torres Strait Islander origin, widowed/divorced/separated, or lived in remote/very remote areas (Table 3). Males had higher odds of multimorbidity using hospital data than with medication data (OR = 1.49 versus OR = 1.07). The age gradient in multimorbidity was more pronounced using administrative data than self-report data (OR >2.5 versus OR = 1.83 for those aged 75–84). People speaking a language other than English at home had 6% higher odds of having multimorbidity (OR = 1.06, 95% CI 1.01–1.10) using medication data and 32% higher odds using hospital data (OR = 1.32, 95% CI 1.22–1.42), but 20% lower odds (OR = 0.80, 95% CI 0.76–0.84) of multimorbidity using self-report data.

**Table 3:** Odds of multimorbidity, by data source.

Variable	n (%)^1^	Self-report data aOR (95% CI)	Medication data aOR (95% CI)	Hospital data aOR (95% CI)	Medication or Hospital data aOR (95% CI)
**Age group**^2^					
45–54 (ref)	8,388 (9.3)	1	1	1	1
55–64	15,830 (17.5)	1.51 (1.43, 1.60)	2.08 (1.94,2.22)	1.93 (1.67,2.23)	2.14 (2.00,2.28)
65–74	35,689 (39.5)	1.56 (1.48, 1.64)	2.73 (2.57,2.91)	1.98 (1.73,2.27)	2.81 (2.65,2.99)
75–84	25,441 (28.2)	1.83 (1.73, 1.93)	4.25 (3.99,4.53)	2.59 (2.26,2.96)	4.53 (4.26,4.81)
85+	5,004 (5.5)	1.62 (1.50, 1.74)	5.13 (4.73,5.55)	3.22 (2.76,3.75)	5.74 (5.31,6.22)
**Sex**^3^					
Female (ref)	50,320 (55.7)	1	1	1	1
Male	40,032 (44.3)	1.27 (1.23, 1.30)	1.07 (1.04,1.10)	1.49 (1.42,1.57)	1.13 (1.10,1.16)
**Remoteness of residence**^4^					
Major city (ref)	37,191 (41.2)	1	1	1	1
Inner regional	33,839 (37.5)	1.06 (1.03, 1.09)	0.97 (0.94,1.01)	0.92 (0.87,0.97)	0.97 (0.94,1.00)
Outer regional	17,506 (19.4)	1.00 (0.96, 1.04)	0.98 (0.94,1.02)	0.85 (0.79,0.91)	0.96 (0.93,1.00)
Remote/very remote	1,803 (2.0)	1.14 (1.03, 1.26)	1.11 (1.00,1.23)	1.28 (1.08,1.53)	1.10 (0.99,1.21)
**Highest education**^4^					
Did not complete school (ref)	42,789 (47.4)	1	1	1	1
High school, apprenticeship, grad dip	35,423 (39.2)	0.95 (0.92, 0.98)	0.80 (0.78,0.83)	0.83 (0.79,0.88)	0.80 (0.78,0.83)
University or higher	9,778 (10.8)	0.90 (0.86, 0.94)	0.65 (0.62,0.68)	0.72 (0.66,0.78)	0.65 (0.62,0.68)
**Aboriginal or Torres Strait Islander** ^4^					
Non Aboriginal (ref)	87,142 (96.5)	1	1	1	1
Aboriginal	904 (1.0)	1.57 (1.38, 1.80)	1.60 (1.39,1.83)	2.09 (1.68,2.61)	1.66 (1.45,1.91)
**Speaks language other than English at home**^4^					
English only (ref)	80,827 (89.5)	1	1	1	1
Other language	9,525 (10.5)	0.80 (0.76, 0.84)	1.06 (1.01,1.10)	1.32 (1.22,1.42)	1.05 (1.00,1.10)
**Country of birth**^4^					
Australia (ref)	66,568 (73.7)	1	1	1	1
Overseas	22,575 (25.0)	0.78 (0.76, 0.81)	0.86 (0.83,0.89)	1.05 (0.99,1.11)	0.86 (0.83,0.89)
**Household incom**^4^					
<20,000 (ref)	35,726 (39.5)	1	1	1	1
20-50k	26,612 (29.5)	0.79 (0.77, 0.82)	0.73 (0.70,0.75)	0.65 (0.61,0.69)	0.72 (0.70,0.75)
50 - 70k	3,298 (3.7)	0.64 (0.59, 0.69)	0.53 (0.49,0.58)	0.49 (0.42,0.58)	0.54 (0.49,0.58)
70k+	1,495 (1.7)	0.58 (0.52, 0.66)	0.49 (0.43,0.55)	0.50 (0.40,0.63)	0.47 (0.42,0.53)
Not stated	15,862 (17.6)	0.75 (0.72, 0.78)	0.85 (0.81,0.88)	0.82 (0.77,0.88)	0.85 (0.82,0.88)
**Marital status**^4^					
Single (ref)	5,774 (6.4)	1	1	1	1
Married/de-facto	58,655 (64.9)	0.88 (0.83, 0.94)	0.96 (0.90,1.02)	0.88 (0.80,0.98)	0.94 (0.89,1.00)
Widowed/divorced/separated	25,246 (27.9)	1.10 (1.03, 1.17)	1.09 (1.02,1.16)	1.09 (0.98,1.22)	1.09 (1.02,1.16)

aOR–odds ratio adjusted for age and sex, unless stated otherwise.

^1^ –percentages do not add up to 100 due to missing data.

^2^ –adjusted for sex only.

^3^ –adjusted for age only.

^4^ –adjusted for age and sex.

### Agreement in multimorbidity between datasets

**Fig 3:**
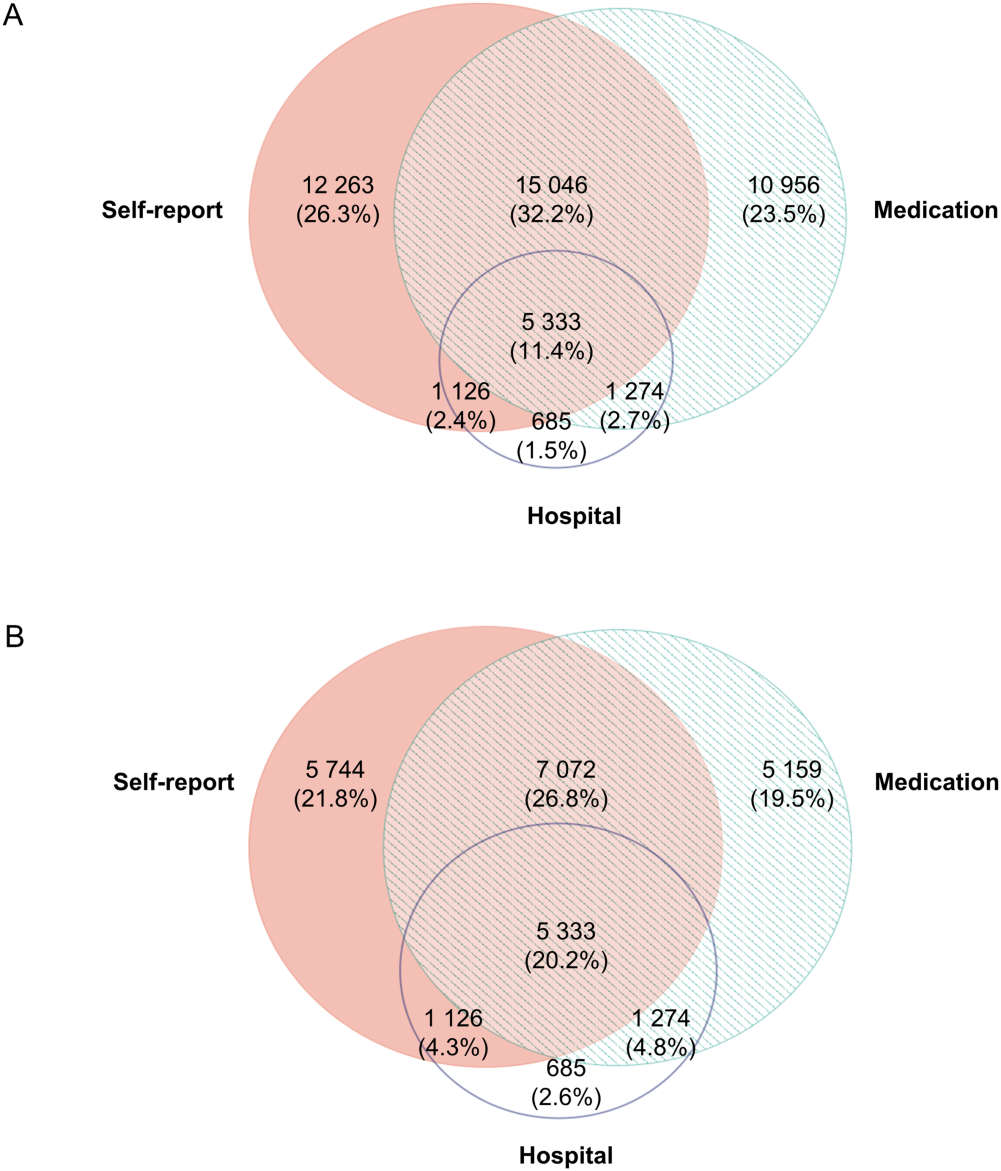
Venn diagram of the prevalence of multimorbidity according to data source. **(A) All data. (B) Hospitalised patients only**. Percentages (%) represent the proportion of all multimorbidity cases ascertained from any of the data sources. Venn diagram constructed using Euler*APE*: http://www.eulerdiagrams.org/eulerAPE/.

A total of 46 683 (52%) people were found to have multimorbidity in any of the three datasets– 33 768 using self-report data, and an additional 12 915 using administrative data only. Of all multimorbid cases, half were identified using a single dataset only, and around one in ten (n = 5 333, 11%) were multimorbid on all three datasets (Fig 3A). When the analyses were restricted to hospitalised patients, the overlap in the datasets increased to 20% (Fig 3B). The agreement on multimorbidity between datasets was poor, with kappa between 0.27 and 0.39, increasing to 0.43 when both hospital and medication data were combined (Table 2).

People identified as being multimorbid in only the self-report data had higher prevalence of cancer, depression, asthma and Parkinson’s disease than those identified only in the administrative datasets. The most common self-report two-way combinations of morbidities were cancer and hypertension (n = 2 177), hypertension and depression (n = 1 243) and a three-way combination of cancer, hypertension and heart disease (n = 376).

Administrative data, however, were more likely to identify hypertension and heart disease than self-report, with the heart disease and hypertension two-way combination being the most prevalent in both medication (n = 7 291) and hospital datasets (n = 323) (data not shown).

## Discussion

This record linkage study of self-report, hospital admission and medication data compared their use for identifying individuals with multimorbidity, based on the most common chronic conditions in Australia. It showed that the ascertainment of multimorbidity varied between data sources, and that, even where the estimated prevalence of multimorbidity was similar for two data sets, the concordance in classification as multimorbid for individual patients was low.

We investigated the level of concordance of identification of eight chronic conditions between self-report and administrative data. We found that chronic conditions identified in hospital data had higher PPVs and low sensitivities, indicating that although the hospital data does not identify all the people with a chronic condition, when such condition is identified, it is generally accurate. Diagnoses may not always be recorded during inpatient episodes of stay, and there is variation in the level or recording between hospitals [10, 11]. In Australia, until recently, there was no mechanism to code diagnoses that do not contribute to hospital stay. Prior to 2015, only diagnoses affecting patient management in a particular episode of care were coded in administrative hospital data. In 2015 codes for temporary use in Australia were assigned to 29 chronic conditions that are present on admission, where the condition does not meet the criteria for coding [52]. We anticipate that this introduction of supplementary codes for chronic conditions will have a positive impact on the sensitivities calculated in the future studies. For studies that do not have supplementary codes, it is advised to incorporate longer lookback periods in order to increase ascertainment of chronic conditions in hospital data [10, 53].

We found that using medication data identifies more cases (higher sensitivity), but at the cost of lower PPV. The lowest PPVs in medication data were found for stroke (16%) and heart disease (35%), the definitions for both of which capture drugs with multiple indications for prescribing. Strong levels of agreement for diabetes, hypertension and Parkinson’s disease are consistent with previous research [41, 54–56], indicating that medication data can potentially be used for capturing these conditions. Low sensitivity and agreement for cancer in our study is congruent with previous Australian studies [54, 57], explained by the fact that chemotherapy drugs are only captured in the PBS data whilst patients are undergoing active treatment. Ascertainment of such cases can be increased by incorporating longer lookback periods. Higher sensitivities for diabetes, hypertension and depression found in our study, compared with a previous Australian study [57], could be attributable to a small sample size in that study, as well as our modified list of depression medications. Namely, we excluded tricyclic antidepressants, as they are commonly prescribed for insomnia and pain. This modification increased our PPV from 55% to 66%.

Selection of the most appropriate set of chronic conditions for other studies will depend on the study’s purpose and the availability of data. Studies requiring accurate case ascertainment should use hospital data (noting that under-ascertainment is likely), or medication data for conditions for which medications are indicated only for that condition (e.g. diabetes) and where there is enough lookback time available. If a comprehensive profile of a patient’s morbidity is needed, we suggest using a combination of data sources in order to increase sensitivity for identifying certain conditions. Caution should be applied when using hospital data for event-based conditions such as stroke, as these may have occurred outside of the time period of data capture, and would thus be under-reported. Identification of stroke patients using medications is also problematic, as the most commonly dispensed medication (Aspirin) is used for a variety of purposes. Furthermore, we recommend caution when interpreting the prevalence of disease or multimorbidity when using a single data source, in line with previously published work [26].

To the best of our knowledge, this is the first study to evaluate the differences in estimates of multimorbidity, using the same list of chronic conditions and the same individuals. Previous data linkage studies have evaluated differences in estimates of chronic disease prevalence within the same individuals [9, 55, 57–60], but did not formally compare case ascertainment of multimorbidity. Pache et al. [24] assessed the prevalence of multimorbidity using three definitions within the same sample, and found that one-third of participants diagnosed with multimorbidity were jointly diagnosed by all three definitions used. In our sample, this estimate was lower (11% - 20%), but this is explained by the smaller number of chronic conditions (8 vs 27), and the standardised list of chronic conditions used in our study, while Pache et al. used a different set of conditions in each of their three definitions,. Van den Bussche et al. [26] used an identical list of chronic conditions in the same setting, albeit among different people, and found that the prevalence of individual chronic conditions was one-third lower in claims data than in primary care data.

The odds of multimorbidity in our study were found to be higher among males, those of older age and those speaking a language other than English at home. The age gradient was noticeable in both hospital and medication datasets, especially with older ages. However, the same gradient was not observed in the self-report data for those aged 85 and over, indicating a possible under-ascertainment of multimorbidity when relying on self-report data only for this age group. Males in our sample had between 7% (PBS data) and 49% (APDC data) higher odds of multimorbidity than females. This is in contrast to other Australian studies, which either found no difference [61] or higher prevalence among females [17], albeit there are differences between the study samples in each of the studies. Compared with the current study, the National Health Survey reported higher prevalence of the most common chronic conditions–hypertension, heart disease and diabetes–among males aged 45 and over [62]. People speaking a language other than English at home in our study were found to have increased odds of having multimorbidity in the administrative data but decreased odds in the survey data. These findings are novel, and have not been reported in the published literature, to the best of our knowledge. A possible explanation is that those speaking another language might have difficulties in understanding medical terminology, which translates to underreporting of conditions in the survey data.

The use of a large-scale cohort study linked with administrative data is a particular strength of our study. This allowed us to use a homogenous population and a common set of chronic conditions to explore ascertainment of multimorbidity using different data sources, which, to the best of our knowledge, has not been done before. Administrative data used in this study are available in most Australian states and territories, allowing replication of results.

Our research has implications for studies examining chronic conditions from a single data source and those examining multimorbidity. We have shown that agreement between self-report and administrative data sources is generally poor, except for a handful of conditions, implying that morbidity and multimorbidity prevalence estimates will vary depending on which data are used. Caution should be applied whenever a single data source is used, taking care to note different levels of capture of chronic disease between data sources. Self-report studies are subject to recall bias, hospitalisation data can only capture conditions for those admitted to hospital and if they are coded during the stay, and medication data may overestimate certain conditions because drugs may have multiple indications. In the case of administrative data, extra care should be taken regarding the time period which is used to ascertain morbidity, with longer times needed to capture more conditions of interest. Choice of which data to use also depends on the purpose of the study. For example, if the aim of the study is to monitor ‘active’ chronic conditions, data linkage of multiple administrative data sources may be more useful than self-report of ever-diagnosis. Furthermore, our study’s finding regarding different individuals, with different combinations of conditions being identified as multimorbid, depending on which datasets are used, poses a challenge when interpreting results of studies examining outcomes of multimorbidity. Careful consideration of individual conditions (which may be under- or over-reported) is needed in order to provide meaningful recommendations for patients with complex care needs.

Although this research generated interesting results, it has some limitations. We based the analyses on a limited set of chronic conditions (arthritis and osteoporosis were notable omissions) available in all three data sources, as well as the available lookback period length. The prevalence of multimorbidity would have been different if a larger set of chronic conditions or a longer lookback period was used. However, all of the conditions used in the current study are National Health Priority Areas [63] as they represent the most common long-term conditions and most commonly managed conditions by GPs [2], significantly contributing to the burden of disease in the Australian community. They are also used in the majority of previously published research [64]. We have used the longest lookback period that the data allowed (2 years), which is longer than the 1-year lookback used in some studies [54, 59].

In the absence of readily available linked primary health care clinical data in Australia, and due to different levels of capture of chronic diseases in administrative datasets, we have used self-report chronic conditions as the reference when examining the concordance between data sets. Although the use of self-report data for identification of chronic disease has been cautioned by some [61], numerous other Australian studies use self-report data to ascertain multimorbidity [17–21]. Validation studies involving participants in the 45 and Up Study found excellent levels of agreement between self-report diabetes [65], country of birth [66] and height and weight [67]. Our data suggest that self-report may be less reliable after the age of 85 and in people speaking a language other than English at home. The use of another data source as a reference could have produced different results.

The use of administrative data poses a different set of challenges. Identification of chronic conditions using APDC data is limited to people who have been admitted to hospital, and having a chronic condition recorded if this was not directly related to the hospital stay, so it is likely to identify only the most severe cases. Medication dispensing information is dependent on the capture of data in the PBS dataset. We were limited to use of PBS-subsidised prescription medicines, which does not include over-the-counter and private prescriptions.

## Conclusions

As administrative data become more widely used for research and evaluation, it is increasingly important to understand their strengths and limitations for ascertaining chronic disease and multimorbidity. This study showed that administrative data has high predictive value for identifying some chronic conditions, but that sensitivity is generally low. Further, it showed that different individuals, with different combinations of conditions, are identified as multimorbid when different data sources are used. Research that explores specific disease combinations and clusters of diseases that commonly co-occur, rather than simple disease counts, is likely to provide more useful insights into the complex care needs of individuals with multiple chronic conditions.

## Supporting information

S1 Fig Construction of study population. APDC–Admitted Patient Data Collection, PBS–Pharmaceutical Benefits Scheme.
(TIF)

S1 Table Morbidities and ICD-10-AM and ATC codes. (DOCX)
