# Supplementary figures and images for: Multimorbidity in Australia: Comparing estimates derived using administrative data sources and survey data
Source: PLoS One. 2017 Aug 29;12(8):e0183817. doi: 10.1371/journal.pone.0183817 (PMC5574547; doi:10.1371/journal.pone.0183817)

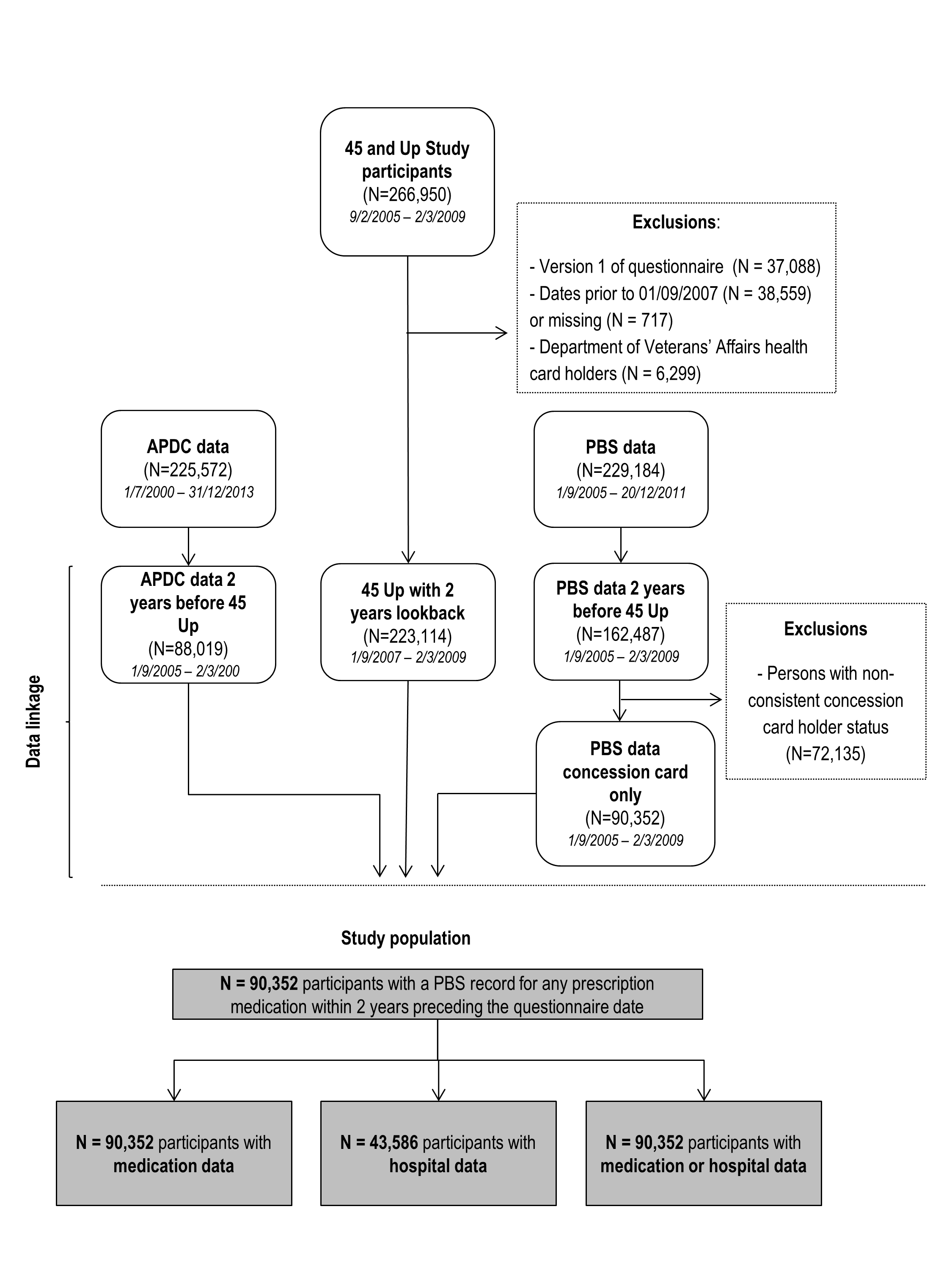

Supplement: S1 Fig — APDC–Admitted Patient Data Collection, PBS–Pharmaceutical Benefits Scheme. (TIF) [file pone.0183817.s001.tif]
