# Supplementary material for: Multimorbidity in Australia: Comparing estimates derived using administrative data sources and survey data
Source: PLoS One. 2017 Aug 29;12(8):e0183817. doi: 10.1371/journal.pone.0183817 (PMC5574547; doi:10.1371/journal.pone.0183817)
Supplement: S1 Table — (DOCX) [file pone.0183817.s002.docx]

| **Chronic condition** | **Medication data** | | **Hospital data** | |
| --- | --- | --- | --- | --- |
|  | **ATC codes** | **Based on** | **ICD-10-AM codes** | **Based on** |
| Cancer | L01AA01, L01AA02, L01AA03, L01AA06, L01AB01, L01AX03, L01BA01, L01BA03, L01BA04, L01BB02, L01BB03, L01BB04, L01BC01, L01BC02, L01BC05, L01BC06, L01CA01, L01CA02, L01CA04, L01CB01, L01CD01, L01CD02, L01DB01, L01DB07, L01DC01, L01XA01, L01XA02, L01XC02, L01XC03, L01XE01, L01XE06, L01XE07, L01XX05, L01XX19, L01XX32 | Rx-Risk-V [35] | C00-C97 (excluding C44) | Charlson Index [32], modified to exclude skin cancer |
| Heart disease | C01DA02, C01DA08, C01DA14, C07AA02, C07AA03, C07AA05, C07AB02, C07AB03, C07AB07, C07AB12, C07AG01, C07AG02, C08CA01, C08CA02, C08CA05, C08CA13, C08DA01, C08DB01 | Olesen et al. [44] At least two classes: α adrenergic blockers, non-loop diuretics, vasodilators, β blockers, calcium channel blockers, renin-antiotensin system inhibitors | I20-I52 |  |
| Hypertension | C02AB01, C02AB02, C02AC01, C02AC05, C02DB02, C02DC01, C02KX01, C02KX02, C02KX03, C03AA03, C03BA04, C03BA11, C03DA01, C03DA04, C03DB01, C03EA01, C09BA02, C09BA04, C09BA06, C09BA09, C09BB02, C09BB05, C09BB10, C09DA02, C09DA04, C09DA06, C09DA07, C09DA08 | Rx-Risk-V [35] | I10, I11-I13, I15 | Elixhauser Index [34] |
| Stroke | B01AA03, B01AB01, B01AB04, B01AC04, B01AC05, B01AC06, B01AC07, B01AC30 | Lix et al. [37] | I60-I64 |  |
| Diabetes | A10AB01, A10AB02, A10AB04, A10AB05, A10AB06, A10AC01, A10AC02, A10AD, A10AD01, A10AD04, A10AE04, A10AE05,  A10B, A10BA02, A10BB01, A10BB07, A10BB09, A10BB12, A10BD02, A10BD03, A10BF01, A10BG02, A10BG03, A10BH01 | Rx-Risk-V [35] | E10-E14 | Elixhauser Index [34]  / Charlson Index [32] |
| Asthma | R03AC02, R03AC03, R03AC12, R03AC13, R03AK06, R03BA01, R03BA02, R03BA05, R03BB01, R03BC01, R03BC03, R03CC02, R03CC03, R03DA04, R03DC03 | Lix et al. [37] | J45, J46 |  |
| Depression | N06AB03, N06AB04, N06AB05, N06AB06, N06AB08, N06AB10, N06AF03, N06AF04, N06AG02, N06AX03, N06AX11, N06AX16, N06AX18, N06AX21 | Rx-Risk-V [35], modified (excluding tricyclic antidepressants) | F32, F33 |  |
| Parkinson's | N04AA01, N04AA02, N04AC01, N04BA02, N04BA03, N04BB01, N04BC01, N04BC02, N04BC05, N04BC06, N04BD01, N04BX02 | Rx-Risk-V [35] | G20, F02.3 |  |
